# Supplementary material for: NEK9 regulates primary cilia formation by acting as a selective autophagy adaptor for MYH9/myosin IIA
Source: Nat Commun. 2021 Jun 2;12:3292. doi: 10.1038/s41467-021-23599-7 (PMC8172835; doi:10.1038/s41467-021-23599-7)
Supplement: Supplementary file 1 — Supplementary Information [file 41467_2021_23599_MOESM1_ESM.pdf]

Supplementary Fig. 1

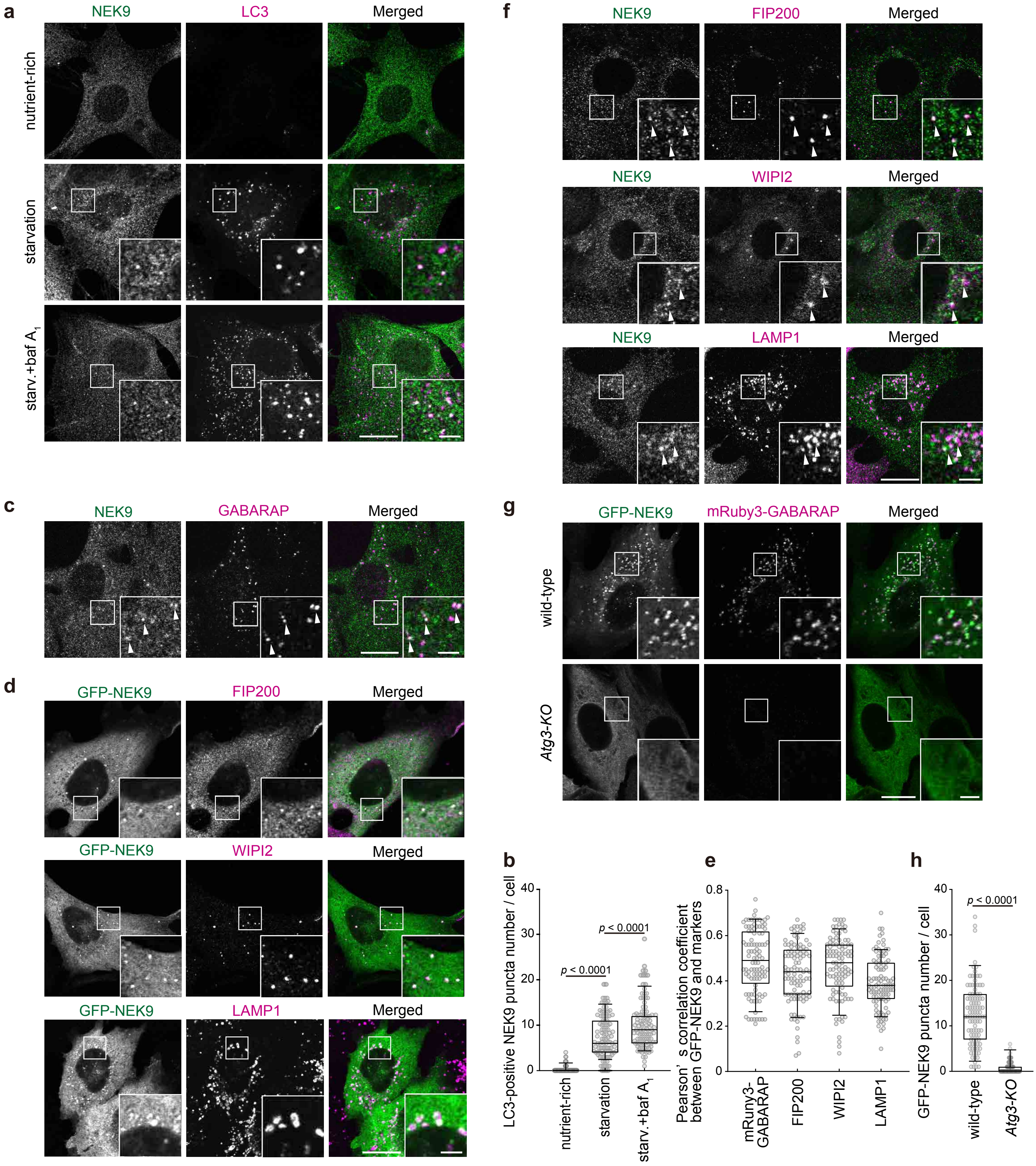

Supplementary Fig. 1 NEK9 associates with autophagic membranes. a, Immunofluorescence microscopy of endogenous NEK9 and LC3 in wild-type MEFs under nutrient-rich conditions or starvation (2 h) with or without 100 nM bafilomycin A_1 (baf A_1). **b**, Quantification of the number of LC3-positive NEK9 puncta in **a**. Data were collected from 100 cells for each condition. **c**, Immunofluorescence microscopy of endogenous NEK9 and GABARAP in wild-type MEFs after starvation (2 h). Data are representative of three biologically independent replicates. **d**, Immunofluorescence microscopy of wild-type MEFs stably expressing GFP-NEK9 after starvation (2 h). Cells were stained with the indicated antibodies. **e**, Colocalization between GFP-NEK9 and mRuby3-GABARAP (Fig. 2a), endogenous FIP200, WIPI2, or LAMP1 in **d** was determined by calculating Pearson's correlation coefficient between intensities within each cell. **f**, Immunofluorescence microscopy of endogenous NEK9 and FIP200, WIPI2, LAMP1 in wild-type MEFs after starvation (2 h). **g**, Immunofluorescence microscopy of wild-type and *Atg3*-KO MEFs expressing GFP-NEK9 and mRuby3-GABARAP after starvation (2 h). **h**, Quantification of the number of GFP-NEK9 puncta in **g**. Data were collected from 100 cells for each condition. Solid bars indicate the medians, boxes the interquartile range (25th to 75th percentile), and whiskers the 10th to 90th percentile in **b**, **e**, and **h**; *p*-values correspond to Tukey's multiple comparisons test in **b**, and to two-tailed Mann–Whitney test in **h**. Scale bars, 10 μm and 3 μm (insets).

Supplementary Fig. 2

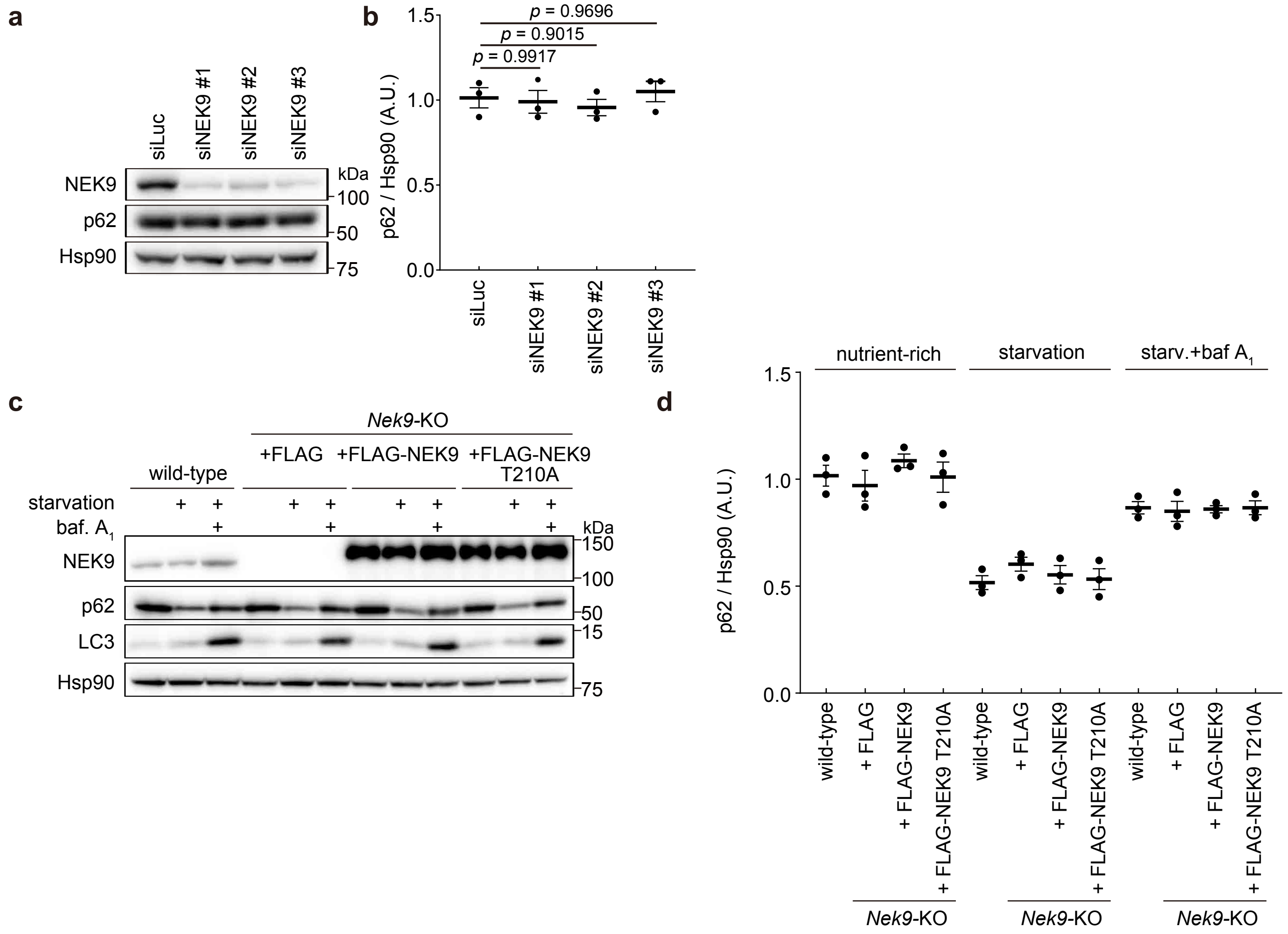

Supplementary Fig. 2 Depletion of NEK9 does not affect selective autophagy of p62. a, Immunoblotting of wild-type HeLa cells in which NEK9 was depleted by siRNA-mediated knockdown. **b**, Quantification of the intensity of the NEK9 bands in **a**. Data represent the mean ± SEM of three independent experiments; *p*-values correspond to a Tukey's multiple comparisons test. **c**, Immunoblotting of wild-type or *Nek9*-KO MEFs stably expressing the indicated constructs under nutrient-rich conditions or after starvation (2 h) with or without 100 nM bafilomycin A_1 (baf A_1). NEK9 T210A, a kinase-dead mutant NEK9. **d**, Quantification of the intensity of the NEK9 bands. Data represent the mean ± SEM of three independent experiments.

Supplementary Fig. 3

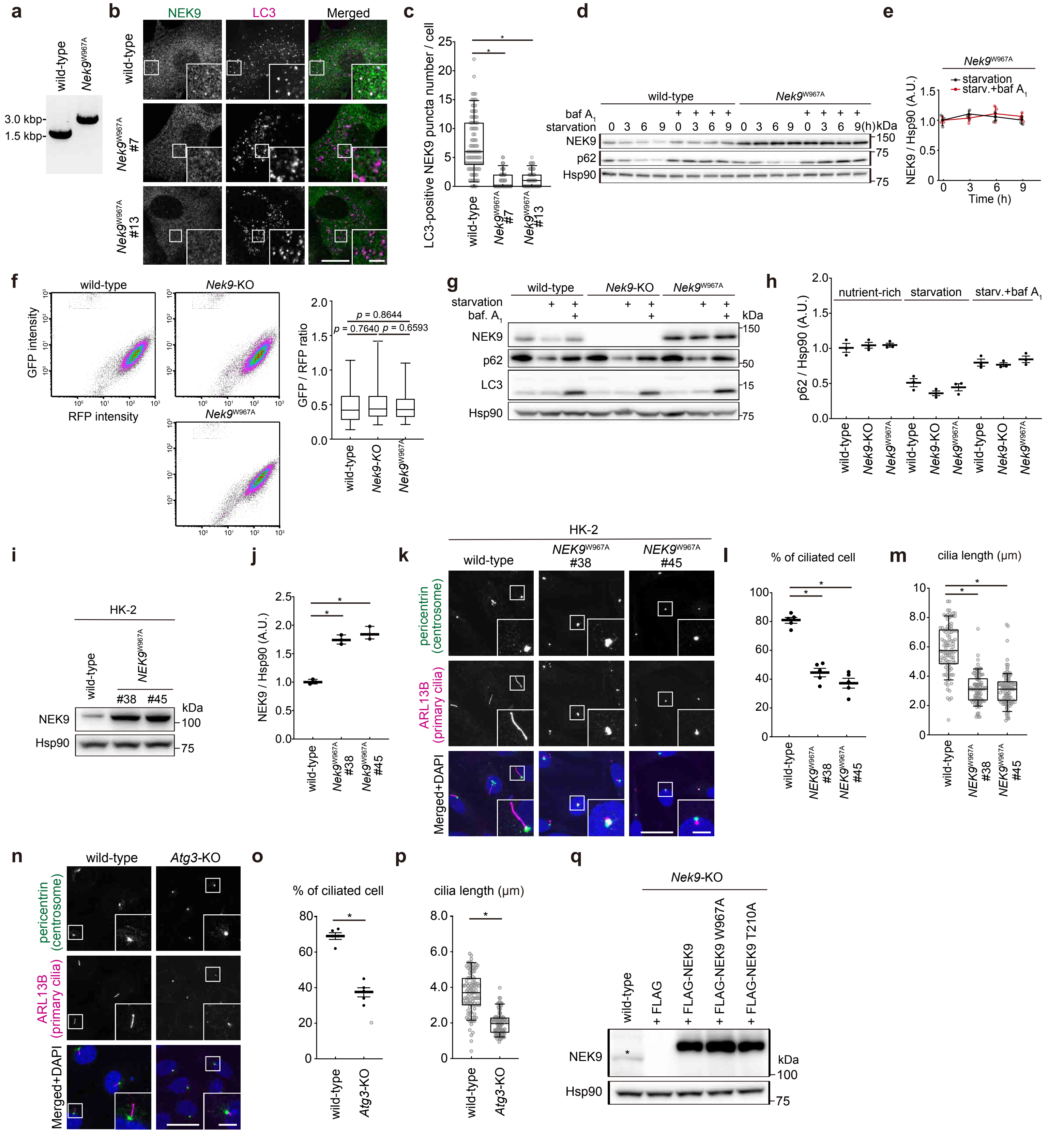

Supplementary Fig. 3 Selective autophagy of NEK9 is required for cilia formation. a, Genotyping of *Nek9*^{W967A} MEFs by PCR. See Fig. 3a for the positions of primers. Data are representative of three biologically independent replicates. **b**, Immunofluorescence microscopy of endogenous NEK9 and LC3 in wild-type or *Nek9*^{W967A} MEFs (two independent clones, #7 and #13) after starvation (2 h). **c**, Quantification of the number of LC3-positive NEK9 puncta in **b**. Data were collected from 100 cells for each cell-type. **d**, Wild-type and *Nek9*^{W967A} (clone #7) MEFs were incubated under starvation conditions with or without 100 nM bafilomycin A_1 for the indicated time. Whole-cell lysates were subjected to immunoblotting. **e**, Quantification of the intensity of the NEK9 bands in **d**. Data represent the mean ± SEM values of three independent experiments. **f**, Quantitative autophagic flux assays. Wild-type, *Nek9*-KO, and *Nek9*^{W967A} (clone #7) MEFs stably expressing the GFP-LC3-RFP reporter were stimulated by Torin 1, an inhibitor of mTOR. The GFP-LC3-RFP reporter is cleaved into GFP-LC3 and RFP by endogenous ATG4 proteases, and GFP-LC3, but not mRuby3, is degraded by autophagy. Accordingly, a reduction in the GFP:RFP ratio represents autophagic flux. Data were collected from 2,000 cells for each cell-type. **g**, Immunoblotting of wild-type, *Nek9*-KO, and *Nek9*^{W967A} (clone #7) MEFs under nutrient-rich conditions or after starvation (2 h) with or without 100 nM bafilomycin A_1 (baf A_1). **h**, Quantification of the intensity of the NEK9 bands. Data represent the mean ± SEM of three independent experiments. **i**, Immunoblotting of wild-type or *NEK9*^{W967A} HK-2 cells (two independent clones, #38 and #45). **j**, Quantification of the intensity of the NEK9 bands in **i**. Data represent the mean ± SEM of three independent experiments. **k,** Immunofluorescence microscopy of wild-type or *NEK9*^{W967A} HK-2 cells. **l**, Frequency of ciliated cells in **k**. Data represent the mean ± SEM of five independent experiments (300 cells were counted in each experiment). **m**, Quantification of cilia length in **k**. Data were collected from 100 ciliated cells for each cell-type. **n**, Immunofluorescence microscopy of wild-type or *Atg3*-KO MEFs. **o**, Frequency of ciliated cells in **n**, as in **l**. Data represent the mean ± SEM of five independent experiments (300 cells were counted in each experiment). **p**, Quantification of cilia length in **n**, as in **m**. Data were collected from 100 ciliated cells for each cell-type. **q**, Immunoblotting of wild-type and *Nek9*-KO MEFs expressing indicated constructs. Asterisk indicates endogenous NEK9 band. In **c**, **f**, **j**, **l**, and **m**, *p*-values correspond to Tukey's multiple comparisons tests and, in **o** and **p**, to two-tailed Mann–Whitney tests; *p <

0.0001. Solid bars indicate the medians, boxes the interquartile range (25th to 75th percentile), and whiskers the 10th to 90th percentile in c, f, m, and p. Scale bars, 10 μm and 3 μm (insets).

Supplementary Fig. 4

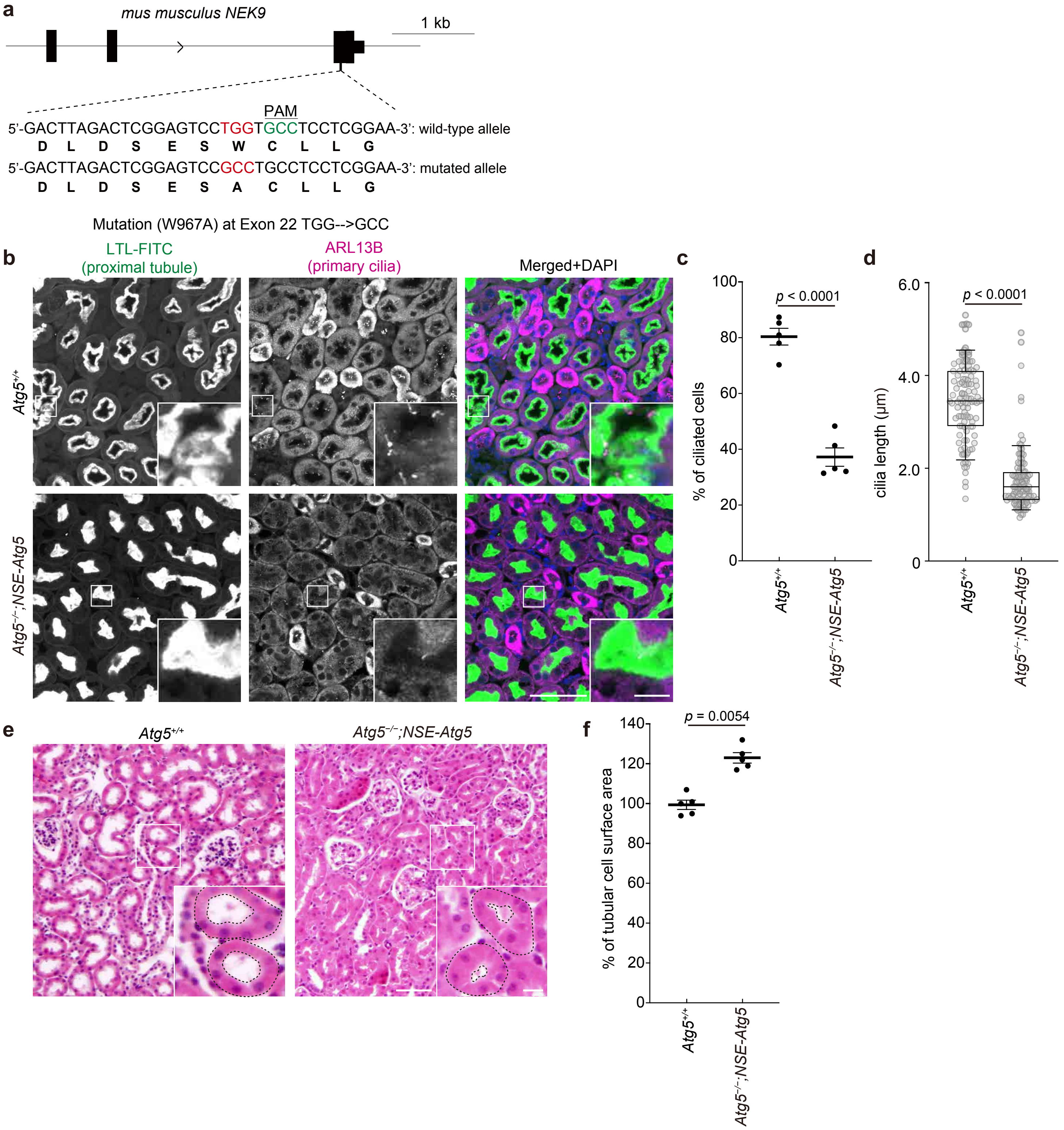

Supplementary Fig. 4 Primary cilia formation is impaired in the kidney in *Atg5*$^{-/-}$*;NSE-Atg5* mice. a, Strategy of the CRISPR-mediated knockin of the W967A mutation at the mouse *Nek9* genomic locus. The protospacer-adjacent motif (PAM) sequence is shown in green. **b**, Immunohistochemistry of the cortical region of kidneys from three-month-old *Atg5*$^{+/+}$ and *Atg5*$^{-/-}$*;NSE-Atg5* mice using LTL-FITC (the lumen of proximal-tubular cells) and anti-ARL13B antibody (primary cilia). Scale bars, 40 μm and 5 μm (insets). **c**, Frequency of ciliated cells in LTL-FITC positive cells in **b**. Data represent the mean ± SEM of three mice (300 cells were counted in each experiment). **d**, Quantification of cilia length in LTL-FITC positive cells in **b**. Data were collected from 100 ciliated cells for each genotype. Solid bars indicate the medians, boxes the interquartile range (25th to 75th percentile), and whiskers the 10th to 90th percentile. **e**, Hematoxylin and eosin staining of the cortical region of kidneys from three-month-old *Atg5*$^{+/+}$ and *Atg5*$^{-/-}$*;NSE-Atg5* mice. Scale bars, 100 μm and 10 μm (insets). **f**, Measurement of the surface area of tubular cells in **e**. Examples of measured areas are shown with broken lines in **e**. Data represent the mean ± SEM of five mice (300 cells were counted in each experiment). In **c**, **d**, and **f**, *p*-values correspond to two-tailed Mann–Whitney tests.

Supplementary Fig. 5

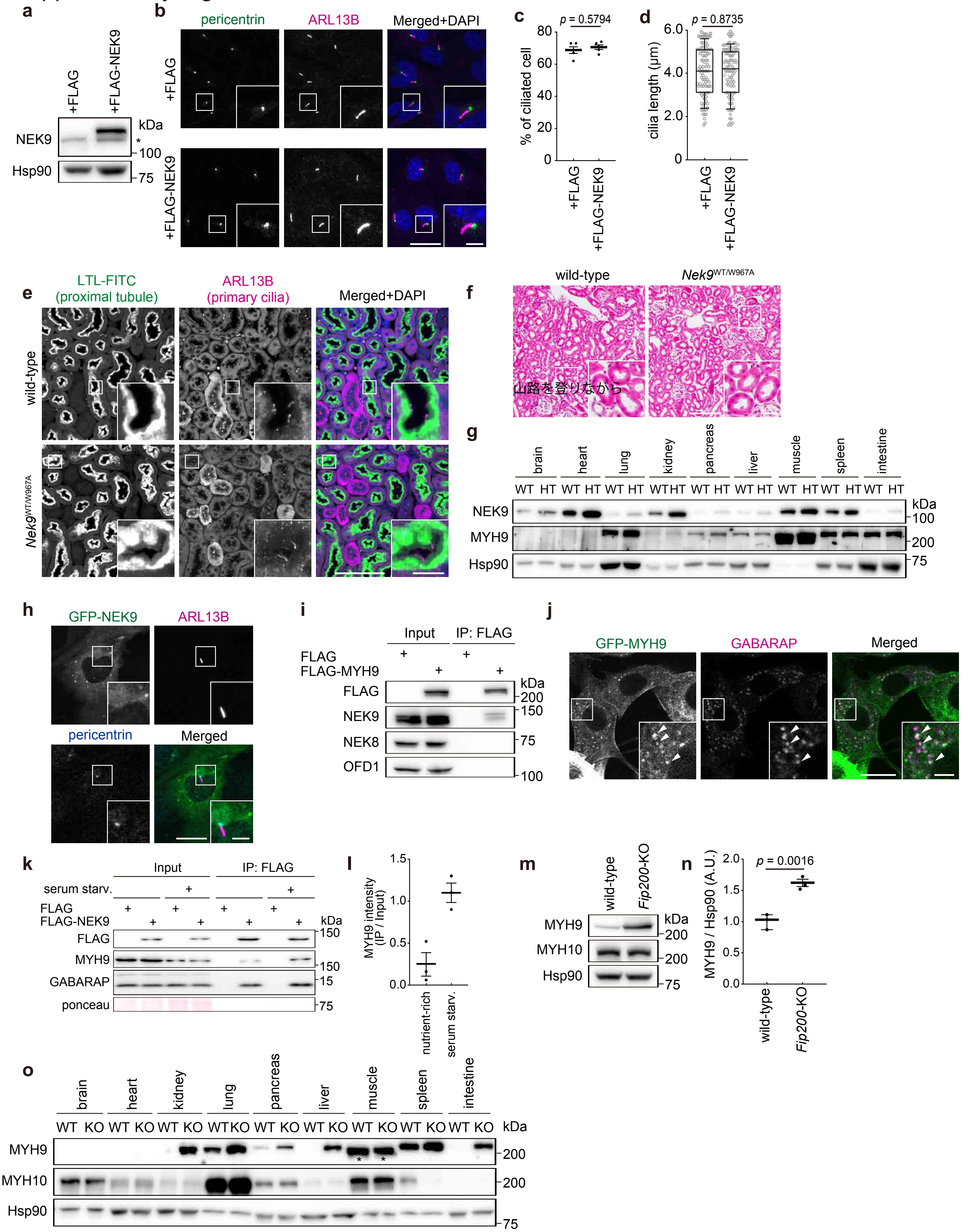

Supplementary Fig. 5 NEK9 is a selective autophagy adaptor for MYH9. a, Immunoblotting of wild-type MEFs stably expressing FLAG or FLAG-NEK9. The asterisk indicates endogenous NEK9. **b**, Immunofluorescence microscopy of wild-type MEFs stably expressing FLAG or FLAG-NEK9 after serum starvation (24 h). Scale bars, 10 μm and 3 μm (insets). **c**, Frequency of ciliated cells in **b**. Data represent the mean ± SEM of five independent experiments (300 cells were counted in each experiment). **d**, Quantification of cilia length in **b**. Data were collected from 100 ciliated cells for each cell-type. Solid bars indicate the medians, boxes the interquartile range (25th to 75th percentile), and whiskers the 10th to 90th percentile. **e**, Immunohistochemistry of the cortical region of kidneys from five-month-old wild-type and heterozygous *Nek9*$^{WT/W967A}$ mice. Data are representative of three biologically independent replicates. Scale bars, 40 μm and 5 μm (insets). **f**, Hematoxylin and eosin staining of the cortical region of kidneys from five-month-old wild-type and *Nek9*$^{WT/W967A}$ mice. Data are representative of three biologically independent replicates. Scale bars, 100 μm and 10 μm (insets). **g**, Immunoblotting of the indicated organs of five-month-old wild-type (WT) and *Nek9*$^{WT/W967A}$ mice (HT). Data are representative of three biologically independent replicates. **h**, Immunofluorescence microscopy of wild-type MEFs stably expressing GFP-NEK9 after serum starvation (24 h), showing that NEK9 does not localize to the centrosome (pericentrin) or cilia (ARL13B). Data are representative of three biologically independent replicates. **i**, Immunoprecipitation using MEFs stably expressing FLAG or FLAG-MYH9. Data are representative of three biologically independent replicates. **j**, Immunofluorescence microscopy of MEFs expressing GFP-MYH9 after serum starvation (2 h). Data are representative of three biologically independent replicates. **k**, Immunoprecipitation using MEFs stably expressing FLAG or FLAG-NEK9 after serum starvation (6 h). **l**, Quantification of the intensity ratio of MYH9 bands (IP / Input) in **k**. Data represent the mean ± SEM of three independent experiments. **m**, Immunoblotting of wild-type or *Fip200*-KO MEFs. **n**, Quantification of the intensity of the MYH9 bands in **m**. Data represent the mean ± SEM of three independent experiments. **o**, Immunoblotting of the indicated organs of three-month-old *Atg5*$^{+/+}$ (WT) and *Atg5*$^{-/-}$*;NSE-Atg5* (KO) mice. Asterisks (*) indicate non-specific bands in skeletal muscles. Data are representative of three biologically independent replicates. In **c**, **d**, **l**, and **n**, *p*-values correspond to two-tailed Mann–Whitney tests. Scale bars, 10 μm and 3 μm (insets).

Supplementary Fig. 6

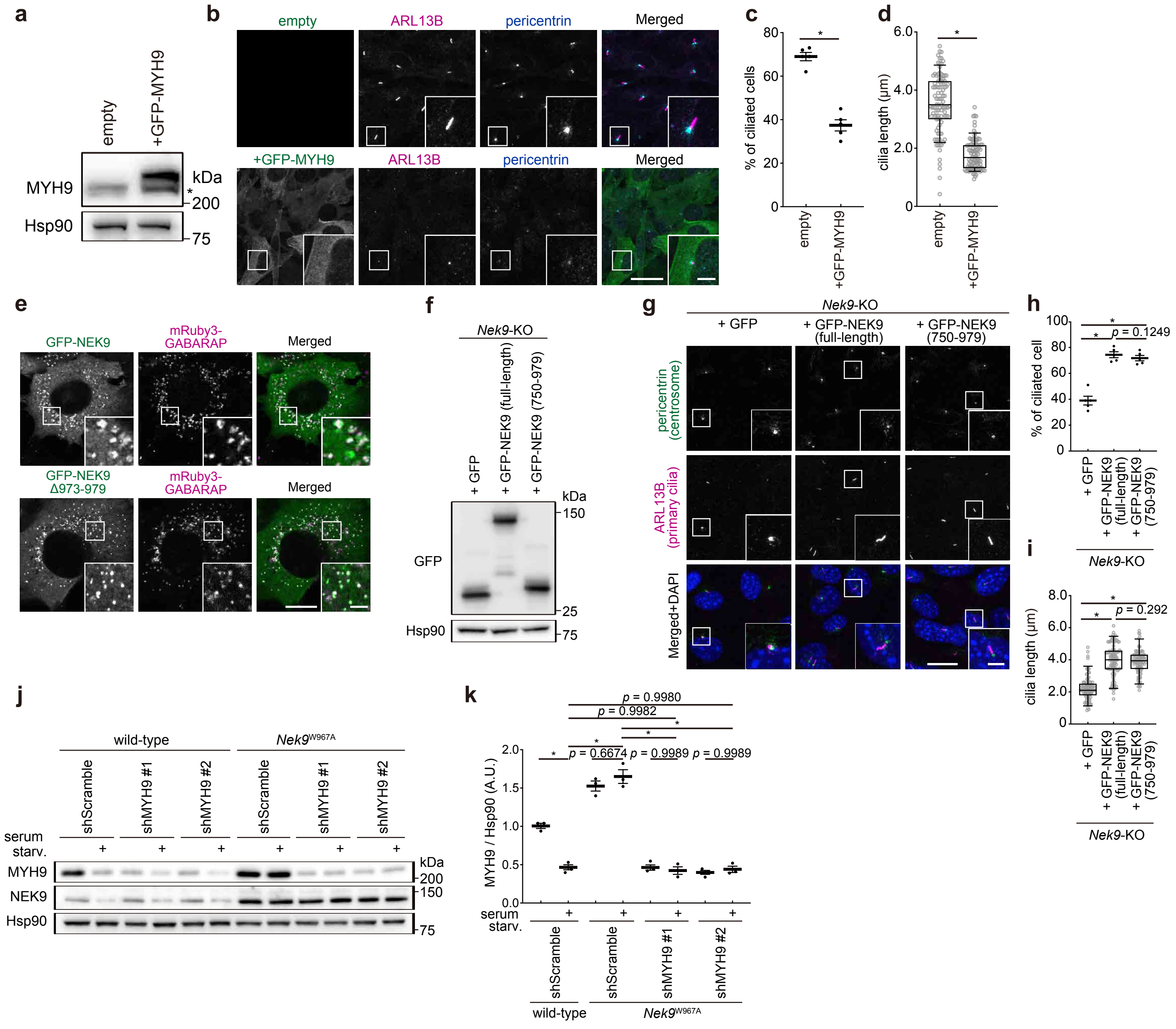

Supplementary Fig. 6 NEK9-mediated selective autophagy of MYH9 is required for primary cilia formation. a, Immunoblotting of wild-type MEFs stably expressing empty-vector or GFP-MYH9. The asterisk indicates endogenous MYH9. Data are representative of three biologically independent replicates. **b**, Immunofluorescence microscopy of wild-type MEFs stably expressing empty-vector or GFP-MYH9 after serum starvation (24 h). **c**, Frequency of ciliated cells in **b**. Data represent the mean ± SEM of five independent experiments (300 cells were counted in each experiment). **d**, Quantification of cilia length in **b**. Data were collected from 100 ciliated cells for each cell-type. **e**, Immunofluorescence microscopy of wild-type MEFs stably expressing GFP-NEK9 or GFP-NEK9 Δ973-979 after serum starvation (4 h). Data are representative of three biologically independent replicates. **f**, Immunoblotting of *Nek9*-KO MEFs expressing indicated constructs. Data are representative of three independent experiments. **g**, Immunofluorescence microscopy of *Nek9*-KO MEFs expressing indicated constructs after serum starvation (24 h). **h,** The frequency of ciliated cells in **g**, as in **c**. Data represent the mean ± SEM of five independent experiments (300 cells were counted in each experiment). **i**, Quantification of cilia length in **g**, as in **d**. Data were collected from 100 ciliated cells for each cell-type. **j**, Immunoblotting of wild-type or *Nek9*^{W967A} MEFs in which MYH9 was depleted by shRNA-mediated knockdown. **k**, Quantification of the intensity of the MYH9 bands in **j**. Data represent the mean ± SEM of three independent experiments. *p*-values correspond to two-tailed Mann–Whitney tests in **c** and **d** and, to a Tukey's multiple comparisons test in **h**, **i**, and **k**; **p* < 0.0001. Scale bars, 10 μm and 3 μm (insets). Solid bars indicate the medians, boxes the interquartile range (25th to 75th percentile), and whiskers the 10th to 90th percentile in **d**, **i**.

Supplementary Fig. 7

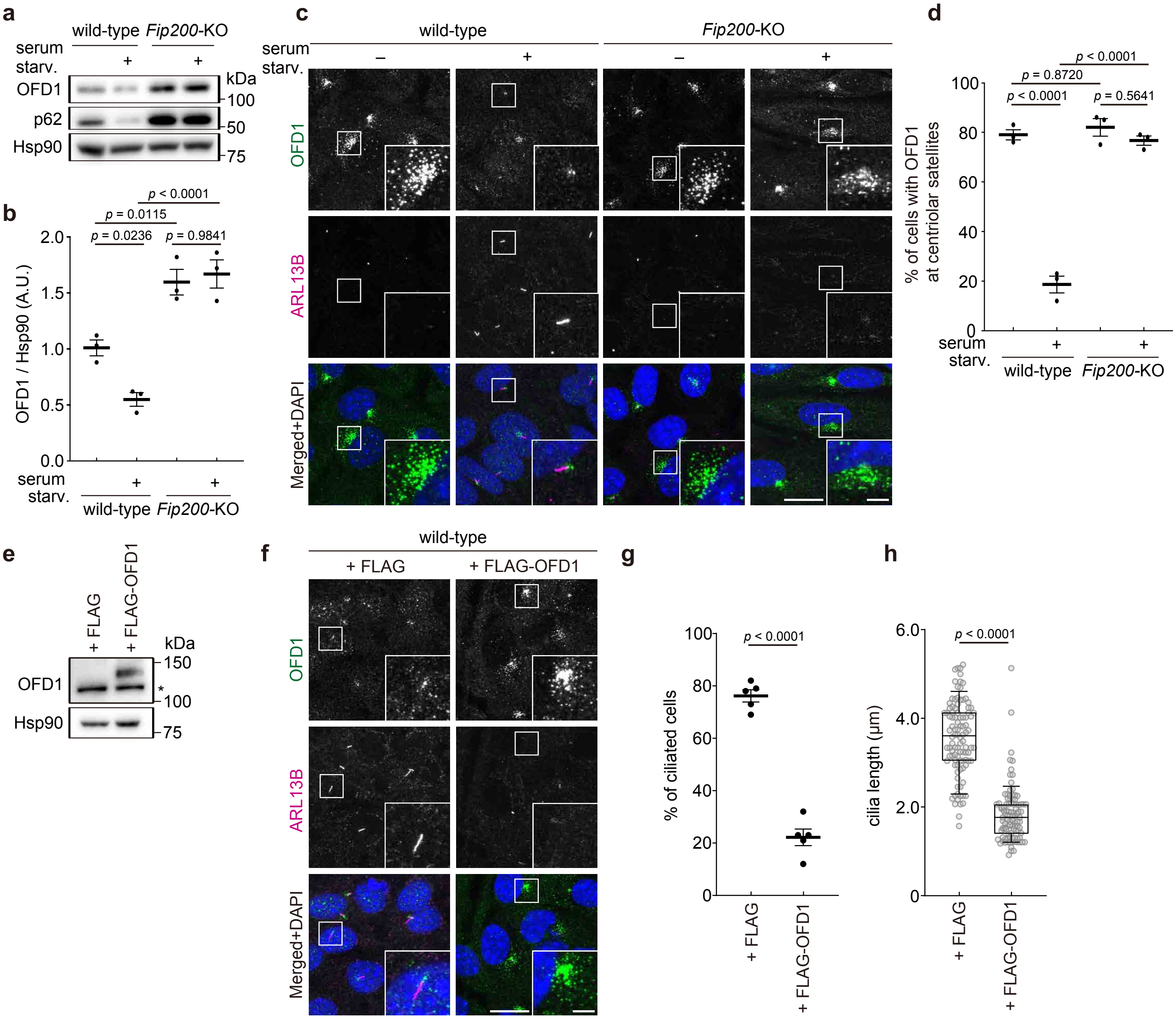

Supplementary Fig. 7 OFD1 at centriolar satellites is a suppressor of ciliogenesis and is degraded by autophagy. a, Immunoblotting of wild-type and *Fip200*-KO MEFs under nutrient-rich conditions or after serum starvation (24 h). **b**, Quantification of the intensity of the OFD1 bands in **a**. Data represent the mean ± SEM of three independent experiments. **c**, Immunofluorescence microscopy of wild-type and *Fip200*-KO MEFs under nutrient-rich conditions or after serum starvation (24 h). **d,** Percentage of cells with centriolar satellites OFD1 in **c**. Data represent the mean ± SEM of three independent experiments (100 cells were counted in each experiment). **e**, Immunoblotting of wild-type MEFs stably expressing FLAG or FLAG-OFD1. The asterisk indicates endogenous OFD1. Data are representative of three biologically independent replicates. **f**, Immunofluorescence microscopy of wild-type MEFs stably expressing FLAG or FLAG-OFD1 after serum starvation (24 h). **g**, Frequency of ciliated cells in **f**. Data represent the mean ± SEM of five independent experiments (300 cells were counted in each experiment). **h**, Quantification of cilia length in **f**. Data were collected from 100 ciliated cells for each cell-type. Solid bars indicate the medians, boxes the interquartile range (25th to 75th percentile), and whiskers the 10th to 90th percentile. In **b** and **d**, *p*-values correspond to Tukey's multiple comparisons tests and, in **g** and **h**, to two-tailed Mann–Whitney tests. Scale bars, 10 μm and 3 μm (insets).

Supplementary Fig. 8

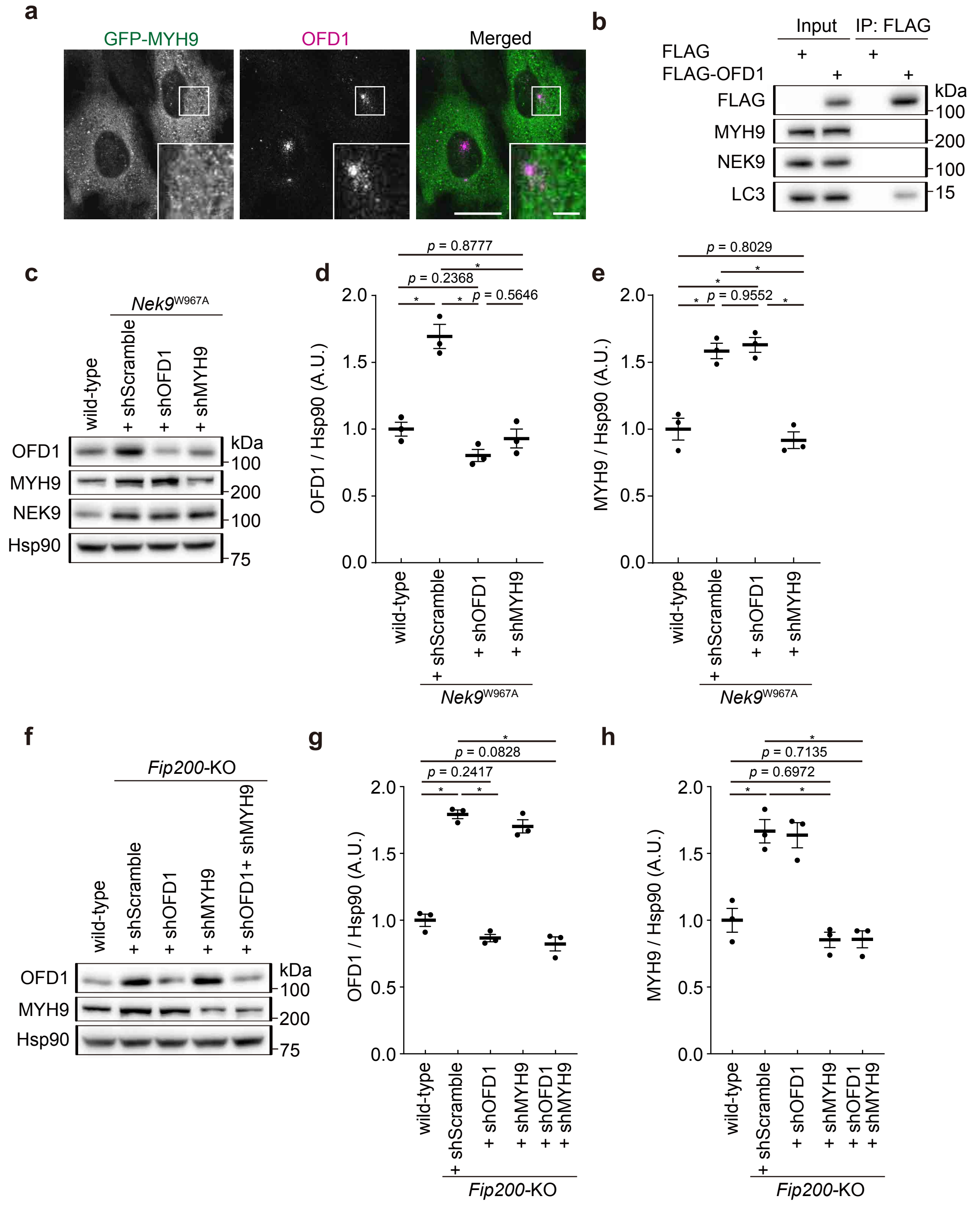

Supplementary Fig. 8 Autophagic degradation of NEK9–MYH9 and OFD1 is required for primary cilia formation. a, Immunofluorescence microscopy of wild-type MEFs expressing GFP-MYH9, showing that MYH9 does not colocalize with OFD1. Scale bars, 10 μm and 3 μm (insets). **b**, Immunoprecipitation using MEFs stably expressing FLAG or FLAG-OFD1. Data are representative of three independent experiments. **c**, Immunoblotting of wild-type and *Nek9*^{W967A} MEFs after serum starvation (24 h). OFD1 or MYH9 was depleted by shRNA-mediated knockdown. **d**, Quantification of the intensity of the OFD1 bands in **c**. **e**, Quantification of the intensity of the MYH9 bands in **c**. **f**, Immunoblotting of wild-type and *Fip200*-KO MEFs after serum starvation (24 h). OFD1 and/or MYH9 were depleted by shRNA-mediated knockdown. **g**, Quantification of the intensity of the OFD1 bands in **f**. **h**, Quantification of the intensity of the MYH9 bands in **f**. Data represent the mean ± SEM of three independent experiments in **d**, **e**, **g**, and **h**; *p*-values correspond to Tukey's multiple comparisons tests; *$p < 0.0001$.
