# Supplementary material for: NEK9 regulates primary cilia formation by acting as a selective autophagy adaptor for MYH9/myosin IIA
Source: Nat Commun. 2021 Jun 2;12:3292. doi: 10.1038/s41467-021-23599-7 (PMC8172835; doi:10.1038/s41467-021-23599-7)
Supplement: Supplementary file 6 — Description of Additional Supplementary Files [file 41467_2021_23599_MOESM6_ESM.docx]

Description of additonal supplemtnaty infomration

Title: Supplementary Data 1

Description; LC-MS/MS analysis of FLAG-GABARAPL1 and FLAGGABARAPL1Y49A/L50A immunoprecipitates.

Title: Supplementary Data 2

Description; LC-MS/MS analysis of FLAG-NEK9 immunoprecipitates.

Title: Supplementary Data 3

Description; List of primers used.
