# Supplementary material for: NEK9 regulates primary cilia formation by acting as a selective autophagy adaptor for MYH9/myosin IIA
Source: Nat Commun. 2021 Jun 2;12:3292. doi: 10.1038/s41467-021-23599-7 (PMC8172835; doi:10.1038/s41467-021-23599-7)
Supplement: Supplementary file 7 — Reporting Summary [file 41467_2021_23599_MOESM7_ESM.pdf]

Corresponding author(s): Noboru Mizushima
Last updated by author(s): Apr 16, 2021

Reporting Summary

Nature Research wishes to improve the reproducibility of the work that we publish. This form provides structure for consistency and transparency in reporting. For further information on Nature Research policies, see our Editorial Policies and the Editorial Policy Checklist.

Statistics

For all statistical analyses, confirm that the following items are present in the figure legend, table legend, main text, or Methods section.

n/a	Confirmed	
☐	☒	The exact sample size (*n*) for each experimental group/condition, given as a discrete number and unit of measurement
☐	☒	A statement on whether measurements were taken from distinct samples or whether the same sample was measured repeatedly
☐	☒	The statistical test(s) used AND whether they are one- or two-sided<br>*Only common tests should be described solely by name; describe more complex techniques in the Methods section.*
☒	☐	A description of all covariates tested
☒	☐	A description of any assumptions or corrections, such as tests of normality and adjustment for multiple comparisons
☐	☒	A full description of the statistical parameters including central tendency (e.g. means) or other basic estimates (e.g. regression coefficient) AND variation (e.g. standard deviation) or associated estimates of uncertainty (e.g. confidence intervals)
☐	☒	For null hypothesis testing, the test statistic (e.g. *F*, *t*, *r*) with confidence intervals, effect sizes, degrees of freedom and *P* value noted<br>*Give P values as exact values whenever suitable.*
☒	☐	For Bayesian analysis, information on the choice of priors and Markov chain Monte Carlo settings
☒	☐	For hierarchical and complex designs, identification of the appropriate level for tests and full reporting of outcomes
☒	☐	Estimates of effect sizes (e.g. Cohen's *d*, Pearson's *r*), indicating how they were calculated

Our web collection on statistics for biologists contains articles on many of the points above.

Software and code

Policy information about availability of computer code

Data collection	Image collection: FV10-ASW (version 04.02, Olympus), FV31S-SW (version 2.4.1.198, Olympus)<br>MS samples of FLAG-GABARAPL1 and FLAG-GABARAPL1Y49A/L50A immunoprecipitates were collected from a Triple TOF 5600+ mass spectrometer (Sciex). All MS/MS spectra were searched against protein sequences of NCBI nonredundant human protein dataset (NCBInr RefSeq Release 71, containing 179,460 entries) using the Protein Pilot software package (Sciex).<br>MS samples of FLAG-NEK9 immunoprecipitates were collected from a Q-Exactive MS instrument (Thermo Fisher Scientific) equipped with a nano HPLC system (Advance UHPLC, Bruker Daltonics) and an HTC-PAL autosampler (CTC Analytics). The obtained raw data were subjected to database search (UniProt, reviewed mouse database as of September 13th, 2018) with Sequest HT algorithm running on Proteome Discoverer 2.2 (Thermo Fisher Scientific).
Data analysis	Image processing: FluoView software (FV10-ASW, version 04.02, or FV31S-SW, version 2.4.1.198, Olympus), Fiji software (Image J, version 1.53c)<br>Statistical analyses: GraphPad Prism 8.0<br>Protein sequence alignment: ClustalW (https://www.ebi.ac.uk/Tools/msa/clustalo/)<br>Domain or secondary structure searching: PSIPRED (http:// bioinf.cs.ucl.ac.uk/psipred/)<br>Phylogenetic tree generation: MEGA-X (version 10.0.5)<br>LIR prediction: iLIR search (https://ilir.warwick.ac.uk/).

For manuscripts utilizing custom algorithms or software that are central to the research but not yet described in published literature, software must be made available to editors and reviewers. We strongly encourage code deposition in a community repository (e.g. GitHub). See the Nature Research guidelines for submitting code & software for further information.

Data

Policy information about availability of data

All manuscripts must include a data availability statement. This statement should provide the following information, where applicable:

- Accession codes, unique identifiers, or web links for publicly available datasets
- A list of figures that have associated raw data
- A description of any restrictions on data availability

The mass spectrometry proteomics data (LC-MS/MS analysis of GABARAPL1 and GABARAPL1Y49A/L50A immunoprecipitates, and FLAG-NEK9 immunoprecipitates) have been deposited to the ProteomeXchange Consortium via the PRIDE partner repository with the dataset identifiers PXD024290 and PXD024292, respectively. Uniprot database search (reviewed mouse database as of September 13th, 2018) was used for analysis of GABARAPL1 and GABARAPL1Y49A/L50A immunoprecipitates. Source data for all graphs and immunoblots of all figures are provided with the paper as Supplementary Information. Source data are provided with this paper.

Field-specific reporting

Please select the one below that is the best fit for your research. If you are not sure, read the appropriate sections before making your selection.

☒ Life sciences ☐ Behavioural & social sciences ☐ Ecological, evolutionary & environmental sciences

For a reference copy of the document with all sections, see nature.com/documents/nr-reporting-summary-flat.pdf

Life sciences study design

All studies must disclose on these points even when the disclosure is negative.

Sample size	The sample size was estimated from preliminary experiments or from previous reports (e.g., Tang et al, Nature volume 502, pages254–257, 2013). No statistical method was used to predetermine sample size.
Data exclusions	No data or animals were excluded from any of the experimental studies presented in the manuscript.
Replication	Confirmed at least three times.
Randomization	Mice were grouped according to genotype, not randomized. In culture cell microscopic experiments, samples from control and experimental groups were randomized in the process of fixation and staining. In immunoblotting analysis, samples were not randomized.
Blinding	Not performed as all groups were treated the same way and there was no analysis of subtle phenotypes.

Reporting for specific materials, systems and methods

We require information from authors about some types of materials, experimental systems and methods used in many studies. Here, indicate whether each material, system or method listed is relevant to your study. If you are not sure if a list item applies to your research, read the appropriate section before selecting a response.

Materials & experimental systems

n/a	Involved in the study	
☐	☒	Antibodies
☐	☒	Eukaryotic cell lines
☒	☐	Palaeontology and archaeology
☐	☒	Animals and other organisms
☒	☐	Human research participants
☒	☐	Clinical data
☒	☐	Dual use research of concern

Methods

n/a	Involved in the study	
☒	☐	ChIP-seq
☒	☐	Flow cytometry
☒	☐	MRI-based neuroimaging

Antibodies

Antibodies used

The following antibodies were used for immunoblotting:
anti-HSP90 (610419; BD)
anti-NEK9 (A301-139A; Bethyl)
anti-NEK8 (A0984; ABclonal)
anti-NEK7 (3057S; Cell Signaling)
anti-MYH9 (A0173; ABclonal)
anti-MYH10 (A12029; ABclonal)
anti-OFD1 (NBP1-89355; Novus Biologicals)

anti-GABARAP (13733S; Cell signaling)
anti-p62/SQSTM1 (PM045; MBL)
anti-GFP (A6455; Thermo Fisher Scientific)
anti-FLAG (F7425; Sigma-Aldrich)
anti-LC3 antibody (M152-3; MBL)
HRP-conjugated anti-mouse IgG (115-035-003; Jackson ImmunoResearch Laboratories)
HRP-conjugated anti-rabbit IgG (111-035-144; Jackson ImmunoResearch Laboratories)
The following antibodies were used for immunocytochemistry and immunohistiochemistry:
anti-NEK9 (sc-100401; Santa Cruz)
anti-FIP200 (17250-1-AP; ProteinTech)
anti-FIP200 (MABC128; Sigma-Aldrich)
anti-WIPI2 (SAB4200400; Sigma-Aldrich)
anti-WIPI2 (MABC91; Sigma-Aldrich)
anti-LAMP1 (ab24170;Abcam)
anti-LAMP1 (ab25630; Abcam)
anti-NEK9 (A301-139A; Bethyl)
anti-OFD1 (NBP1-89355; Novus Biologicals)
anti-pericentrin (abcam; 4448)
anti-LC3 (CTB-LC3-2-IC; CosmoBio)
anti-ARL13B (abcam; 136648)
Alexa Fluor 488-conjugated goat anti-mouse IgG (A-11029; Thermo Fisher Scientific)
Alexa Fluor 568-conjugated goat anti-rabbit IgG (A-11036; Thermo Fisher Scientific)
Alexa Fluor 660-conjugated goat anti-mouse IgG (A-21055; Thermo Fisher Scientific)

Validation

Validation statements are available from manufacturers:
The antibodies used for immunoblotting:
anti-HSP90 (610419; BD; https://www.bdbiosciences.com/eu/applications/research/apoptosis/purified-antibodies/purified-mouse-anti-hsp90-68hsp90/p/610419)
anti-NEK9 (A301-139A; Bethyl; https://www.bethyl.com/product/A301-139A/NEK9+Antibody)
anti-NEK8 (A0984; ABclonal; https://abclonal.co.jp/catalog-antibodies/NEK8RabbitpAb/A0984)
anti-NEK7 (3057S; Cell Signaling; https://www.cellsignal.com/products/primary-antibodies/nek7-c34c3-rabbit-mab/3057?Ntk=Products&Ntt=3057)
anti-MYH9 (A0173; ABclonal; https://abclonal.com/catalog-antibodies/MYH9PolyclonalAntibody/A0173)
anti-MYH10 (A12029; ABclonal; https://abclonal.com/catalog-antibodies/MYH10PolyclonalAntibody/A12029)
anti-OFD1 (NBP1-89355; Novus Biologicals; https://www.novusbio.com/products/ofd1-antibody_nbp1-89355)
anti-GABARAP (13733S; Cell signaling; https://www.cellsignal.com/products/primary-antibodies/gabarap-e1j4e-rabbit-mab/13733?Ntk=Products&Ntt=13733)
anti-p62/SQSTM1 (PM045; MBL; https://www.mblintl.com/products/pm045/)
anti-GFP (A6455; Thermo Fisher Scientific; https://www.thermofisher.com/antibody/product/GFP-Antibody-Polyclonal/A-6455)
anti-FLAG (F7425; Sigma-Aldrich; https://www.sigmaaldrich.com/catalog/search/ProductDetail/SIGMA/F7425)
anti-LC3 (M152-3; MBL; https://www.mblintl.com/products/m152-3ms/)
HRP-conjugated goat anti mouse IgG (115-035-003; Jackson ImmunoResearch Laboratories)
HRP-conjugated anti-rabbit IgG (111-035-144; Jackson ImmunoResearch Laboratories)
The following antibodies were used for immunocytochemistry and immunohistiochemistry:
anti-FIP200 (17250-1-AP; ProteinTech; https://www.ptglab.com/products/RB1CC1-Antibody-17250-1-AP.htm#)
anti-WIPI2 (SAB4200400; Sigma-Aldrich; https://www.sigmaaldrich.com/catalog/product/sigma/sab4200400?lang=en&region=US)
anti-LAMP1 (ab24170;Abcam; https://www.abcam.com/lamp1-antibody-lysosome-marker-ab24170.html)
anti-NEK9 (A301-139A; Bethyl; https://www.bethyl.com/product/A301-139A/NEK9+Antibody)
anti-OFD1 (NBP1-89355; Novus Biologicals; https://www.novusbio.com/products/ofd1-antibody_nbp1-89355)
anti-pericentrin (abcam; 4448; https://www.abcam.com/pericentrin-antibody-centrosome-marker-ab4448.html)
anti-LC3 (CTB-LC3-2-IC; CosmoBio; https://www.cosmobiousa.com/products/anti-lc3-mab-clone-lc3-1703)
anti-ARL13B (abcam; 136648; https://www.abcam.com/arl13b-antibody-n295b66-ab136648.html)
Alexa Fluor 488-conjugated goat anti-mouse IgG (A-11029; Thermo Fisher Scientific; https://www.thermofisher.com/antibody/product/Goat-anti-Mouse-IgG-H-L-Highly-Cross-Adsorbed-Secondary-Antibody-Polyclonal/A-11029)
Alexa Fluor 568-conjugated goat anti-rabbit IgG (A-11036; Thermo Fisher Scientific; https://www.thermofisher.com/antibody/product/Goat-anti-Rabbit-IgG-H-L-Highly-Cross-Adsorbed-Secondary-Antibody-Polyclonal/A-11036)
Alexa Fluor 660-conjugated goat anti-mouse IgG (A-21055; Thermo Fisher Scientific; https://www.thermofisher.com/antibody/product/Goat-anti-Mouse-IgG-H-L-Highly-Cross-Adsorbed-Secondary-Antibody-Polyclonal/A-21055)

Eukaryotic cell lines

Policy information about cell lines

Cell line source(s)

HeLa and HEK293T cells were bought from RIKEN (RCB0007 and RCB2202, respectively)
Fip200 +/+ and Fip200-/- MEFs were provided by Jun-lin Guan, University of Cin-Cinnati
Atg3 +/+ and Atg3 -/- MEFs were provided by Masaaki Komatsu, Juntendo University
HK-2 cells were obtained from ATCC (Number: SCRC-2190™)

Authentication

HeLa and HEK293T cells were validated by STR profiling at RIKEN.
HK-2 cells from ATCC were validated by STR profiling, visual inspection, and careful maintainenance in a central lab cell bank.

Mycoplasma contamination

The mammalian cell lines were confirmed to be negative for mycoplasma contamination by PCR.

Commonly misidentified lines (See ICLAC register)	No commonly misidentified cell lines were used.

Animals and other organisms

Policy information about studies involving animals; ARRIVE guidelines recommended for reporting animal research

Laboratory animals	Mice (Mus musculus) were on C57BL/6J genetic background. There was no bias between male and female mice used in our study. Wild-type or mutant neonatal mice were prepared by mating male and female mice at 2-10 months of age. We analyzed five-month-old Nek9W967A/W967A or Nek9WT/W967A mice and three-month-old Atg5−/−;NSE-Atg5 mice for analysis of the kidney. All mice were housed in a specific pathogen-free room maintained at a constant ambient temperature of 22-26 degree Celsius, 40-65% of humidity under a 12 h light/dark cycle with free access to food and drink.
Wild animals	This study did not involve wild animals.
Field-collected samples	This study did not involve field collected samples.
Ethics oversight	All experimental procedures and treatments were conducted in compliance with the Institutional Animal Care and Use Committee of the University of Tokyo (Medical-P17-084) and the Animal Care and Use Committee of the National Institute of Quantum and Radiological Science and Technology (1610111 and 1610121).

Note that full information on the approval of the study protocol must also be provided in the manuscript.
